# Advances in Diagnosis and Pathophysiology of Microvascular Dysfunction

**DOI:** 10.3390/diagnostics12030620

**Published:** 2022-03-02

**Authors:** Yih-Kuen Jan

**Affiliations:** Department of Kinesiology and Community Health, University of Illinois at Urbana-Champaign, Champaign, IL 61820, USA; yjan@illinois.edu

Microcirculation is the system that brings oxygen and nutrients to local cells and removes metabolic wastes. The study of the structure, function, and adaptation of the microvascular system plays an essential role in understanding cardiovascular diseases [1]. Research studies have shown that the use of dynamics of microvascular dysfunction is promising as an indicator and risk determinant of other diseases, including diabetes, cancer, and stroke [1,2]. Additionally, the microvasculature is recognized as one of the most important routes for drug delivery in disease conditions [1,3]. Recent advances in computational models and genomic technologies have provided new opportunities to investigate microvascular dysfunction and its role in various diseases. This Special Issue highlights recent advances in the diagnosis and pathophysiology of microvascular dysfunction. 

Under pathophysiological conditions (e.g., diabetes, sickle cell anemia, and central retinal vein occlusion), structural and functional adaptations of microvascular networks and erythrocytes can be quantified using novel imaging technologies and advanced quantification metrics, such as multispectral and hyperspectral imaging and optical coherence tomography. Asaro and Cabrales [4] propose a novel paradigm to induce red blood cell (RBC) vesiculation during vascular flow of red blood cells adhering to the vascular endothelium and to the red pulp of the spleen. They hypothesize that erythrocytes can be driven to vesiculate by adhering to endothelial splenic slits via tether formation. Using simulation results of red cell deformability in the vesiculation process, their synthesized findings provide a mechanistic basis for membrane loss and the formation of lysed RBCs in the spleen. Asaro and Cabrales [4] also discuss how various diseases and aging affect RBC adherence to endothelial cells, including diabetes, Gaucher disease, and myeloproliferative neoplasms. However, these mechanistic approaches using the combined RBC characteristics of deformability and adhesion allow for the early detection and diagnosis of diseases.

Macular degeneration is one of the most popular applications of microvascular research. Shu et al. [5] investigated the association of autophagy-related gene expression with age-related macular degeneration (AMD). mRNA was assessed by real-time polymerase chain reaction (RT-PCR) to evaluate whether the expression of 26 autophagy-related genes (ATGs) was correlated with AMD. Their results showed that both the neovascular AMD (nAMD) and polypoidal choroidal vasculopathy (PCV) groups had significantly higher mRNA levels of gamma-aminobutyric acid receptor-associated protein-like 1 (GABARAPL1) and microtubule-associated proteins 1A/1B light chain 3B (MAP1LC3B) than the control group. They demonstrated the possibility of assessing autophagy-related gene expression by conjunctival impression cytology. 

Skin is the most accessible organ [3]. Research studies have shown promising evidence that the cutaneous microcirculation can be used as a surrogate to assess underlying diseases [6]. Chia et al. [7] used laser Doppler flowmetry (LDF) to assess the characteristics of shoulder microcirculation abnormality in workers with myofascial pain. Their results showed that the shoulder pain level was significantly higher in the patient than in the control group. Their study provides initial evidence of the use of LDF to diagnose myofascial pain. 

The microvascular dynamics assessed by LDF provides a novel way to diagnose the underlying mechanisms of microvascular dysfunction caused by the endothelial, neurogenic, and myogenic controls [8]. Bau et al. [9] used this method to examine whether a vascular impairment is associated with chronic muscle pain and how an intervention (transverse friction massage) could improve the vascular impairment. Their results show that the differences in the baseline blood flow between the asymptomatic and patient groups were non-significant; however, the standard deviations in the measurements of the upper trapezius muscle in the patients were significantly larger. Their results also show that this intervention could significantly improve blood flow to the treated area.

Finally, Nikolov and Popovski [10] review the role of the matrix metalloproteinase (MMP) family in vascular remodeling. MMP-2 and MMP-9 are associated with collagen degradation. MMP-2 is capable of cleaving gelatine and types I and IV collagens, while MMP-9 is incapable of direct proteolysis of collagen I and digests collagen type IV. MMP-2 and -9 are both important regulators of vascular and uterine remodeling in healthy pregnancy. Their review discusses the role of MMP-2 and MMP-9 as markers for diagnosis, prognosis, and monitoring of preeclampsia development. 

This Special Issue elucidates some new aspects of microvascular dysfunction as well as novel applications using microvascular dysfunction as the disease state. These new studies provide new insights into microvascular dysfunction as well as its potential role in diagnosing various diseases. 
